# Norovirus GII.4 Virus-like Particles Recognize Galactosylceramides in Domains of Planar Supported Lipid Bilayers**

**DOI:** 10.1002/anie.201205972

**Published:** 2012-10-24

**Authors:** Marta Bally, Gustaf E Rydell, Raphael Zahn, Waqas Nasir, Christian Eggeling, Michael E Breimer, Lennart Svensson, Fredrik Höök, Göran Larson

**Affiliations:** Department of Applied Physics, Chalmers University of TechnologySE-41133 Göteborg (Sweden); Department of Clinical Chemistry and Transfusion Medicine, Sahlgrenska Academy, University of Gothenburg(Sweden); Traffic, Signaling, and Delivery Laboratory, Centre de Recherche, Institut CurieCNRS UMR144 (France); Department of Surgery, Sahlgrenska Academy, University of Gothenburg(Sweden); Laboratory of Biosensors and Bioelectronics, Institute of Biomedical Engineering, Universität Zürich und ETH Zürich(Switzerland); Max-Planck-Institut für Biophysikalische Chemie (Germany), Currently: WIMM, University of Oxford(UK); Division of Molecular Virology, University of Linköping(Sweden)

**Keywords:** galactosylceramide, glycolipids, noroviruses, supported lipid bilayer, viruses

In-depth understanding of the cell-surface dependent processes leading to virus binding and infection of host cells, including the identification of new receptors mediating the initial steps, is of central importance for the development of new anti-viral therapies. One still poorly understood but highly contagious virus is the human norovirus, which causes acute gastroenteritis.^1^ It is a single-stranded non-enveloped RNA virus belonging to the *Caliciviridae* family and during recent years, new strains, particularly of the genotype GII.4, have emerged causing worldwide epidemics.^2^ However, detailed knowledge on 1) the target cell, 2) the mechanism of viral entry, and 3) the receptor(s) for cellular uptake are still lacking.

Because there is presently no simple cell-culture model available for propagating human norovirus, experimental studies aimed at resolving (1) to (3) are heavily dependent on in vitro production of virus-like particles (VLPs),^3^ which, for the norovirus, form spontaneously when the major capsid protein (VP1) is recombinantly expressed.^2^ Such VLPs show a symmetric icosahedral shell assembled from 180 copies of the VP1 protein,^4^ with a morphology, antigenicity, and binding characteristics that reflect those of native viruses.^5^ VLP binding studies have revealed that most human noroviruses recognize, in a strain-specific manner, ABO, Lewis (*FUT3*), and secretor (*FUT2*) gene-dependent histo-blood group antigens (HBGAs),^1^, ^6^ on glycoproteins and glycosphingolipids.6c, 7 VLPs also bind in a fucose-dependent manner to epithelial cells of duodenal tissue sections and saliva from secretors but not from non-secretors.^8^

This study was motivated by the fact that histopathological biopsies point to the upper small intestine as the likely target organ for this virus.^9^ Besides type 1 chain fucolipids, epithelial cells of the human small intestine contain large amounts of monoglycosylceramides (mainly β-galactosyl-ceramide, (GalCer)).^10^ Moreover, although not previously explored in the context of norovirus infection, GalCer and other short-chain glycosphingolipids (GSLs) have been identified as facilitators of other viral infections, such as HIV.^11^ To explore if GalCer could potentially be a receptor for norovirus infection, thus acting alone or in combination with other HBGAs on glycolipids that have been identified to interact specifically with different norovirus strains,6c, 7 this work is focused on characterizing norovirus VLP binding to GalCer.

GSLs are usually found together with cholesterol and sphingomyelin in phase-separated domains.^12^ In addition to the verified role of GSL domains in a variety of cellular processes,12b, 13 there is also increasing evidence that such domains can be sites for pathogen attack.^14^ Of particular relevance to this work, GalCer is believed to cluster into microdomains, as it has been isolated in the form of detergent-resistant membranes.^15^ Phase separation of GalCer is further supported by a variety of studies on model supported lipid bilayers (SLBs).^16^ Herein, we report the recognition of GalCer by norovirus VLPs, with particular focus on the dependence of this interaction on GSL presentation and packing, including domain formation.

Protein–carbohydrate interactions between the VLPs from norovirus and GalCer were identified using the chromatogram binding assay (CBA), a well-established method for probing binding of proteins to GSLs.^17^ As shown in Figure 1, VLPs bound to reference H type 1 GSLs, typically present in the small intestine of an OLe(a-b-) secretor but not of an OLe(a+b-) non-secretor, a phenotype known to be genetically resistant to infection by most GII.4 norovirus strains.^18^ Interestingly, VLPs also bound to monoglycosylceramides, a mixture of GlcCer and GalCer, of small- and large-intestinal samples of both secretors and non-secretors, as well as to purified galactosylceramides of human meconium. The two bands of galactosylceramides are related to heterogeneity of the ceramides with both dihydroxylated sphingosine (d18:1) and trihydroxylated phytosphingosine (t18:0) present together with a span of hydroxylated (hC16:0–hC24:0) fatty acids.^19^ In control experiments, the VLPs bound equally well to monospecies (greater than 95 %) galactosylceramides with d18:1–16:0 or d18:1–24:1 ceramides. VLP binding to GalCer was not previously reported, whereas binding to HBGAs was in agreement with previous studies on GSLs6c, 7, 9 and on neoglycoproteins.^20^

**Figure 1:**
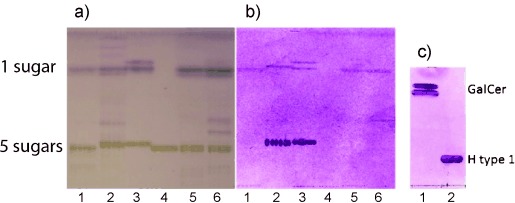
Thin-layer chromatogram binding assay showing binding of Dijon GII.4 VLPs to GSLs. a) Chemical staining with a predominance of GSL with 1 and 5 sugar residues (indicated at the left). b) VLPs binding to specific GSLs. The GSL samples were total non-acid GSLs from human small-intestinal epithelial cells from a blood group OLe(a+b-)non-secretor (*FUT2−/−*; lane 1), an OLe(a-b-)secretor (*FUT2+/±*; lane 2), pure GalCer (lane 3, upper bands), blood group H type 1 chain pentaglycosylceramide (lane 3, lower band), blood group Le^a^ pentaglycosylceramide (lane 4), and total non-acid GSLs from human large-intestine epithelial cells of an OLe(a-b+)secretor (lane 5) and from a mucosa scraping of an ABLe(a+b-)non-secretor (lane 6). c) Binding of Dijon VLPs to GalCer (lane 1) and H type 1 chain GSL (lane 2) on a separate TLC chromatogram.

While the CBA offers the possibility to screen, in a standardized manner, for interactions between the GSLs and the virus particles, the ligand presentation on a TLC plate is likely to differ significantly from the one in a natural lipid membrane. Because of the positive interaction between the norovirus VLPs and GalCer, we extended the investigation to include VLP binding to GalCer mixed with 1-palmitoyl-2-oleoyl-*sn*-glycero-3-phosphocholine (POPC) lipids in planar SLBs. Such model cell membranes make it possible to present the ligand to the virus particle in a more native-like environment while preserving relevant characteristics such as membrane fluidity, ligand mobility, and the ability of GSLs to organize into microdomains.^21^

SLBs containing native GalCer were produced by surface-induced fusion of lipid vesicles on SiO_2_ substrates at 37 °C, that is, above the phase-transition temperature of GalCer. SLBs containing 10 % (*w*/*w*) GalCer were characterized by atomic force microscopy (AFM), which permits high-resolution imaging of individual GalCer-rich domains at different temperatures, down to a few hundred nanometers. At 37 °C, the SLBs were mostly homogeneous and defect-free; the domain surface coverage being less than 0.4 % (Figure 2 a). Cooling across the miscibility gap leads to phase separation and at 22 °C, approximately 7 % of the surface was covered with micrometer-sized domains (average area: 21 μm^2^; Figure 2 b). As shown in Figure 2 c, these domains could be clearly visualized as topographically higher fractal structures (height: approximately 1.5 nm), in good agreement with other studies.^16^, ^22^

**Figure 2:**
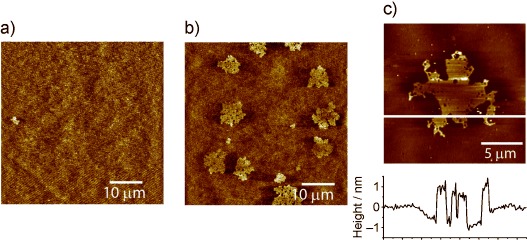
AFM images of POPC bilayers containing 10 % GalCer. a) At 37 °C, the bilayer is homogeneous with few domains (less than 0.4 %); b) at 22 °C, the average domain surface coverage is 7 %. c) Magnification of a fractal GalCer domain with corresponding height profile (white line in the image).

A quartz crystal microbalance with dissipation monitoring (QCM-D)^23^ was used to investigate the influence of microdomains on norovirus binding to GalCer. This in situ technique is based on an oscillating piezoelectric quartz crystal resonator. A negative change in resonance frequency (Δ*f*) is, to a good approximation, proportional to the bound mass, including the water molecules hydrodynamically coupled to the film. Additionally, the damping of the crystal (recorded simultaneously as a change in the energy dissipation (Δ*D*)) provides information on the viscoelastic and structural properties of the film: the softer the film, the higher the Δ*D* values. The formation of the bilayer at 37 °C was verified in situ (Figure 3, time<0): the initial vesicle adsorption resulted in a decrease in Δ*f* and an increase in Δ*D*. When a critical vesicle surface coverage was reached, this was followed by a spontaneous increase and decrease in Δ*f* and Δ*D* respectively, which is characteristic for vesicle rupture and fusion into a more rigid (Δ*D*=0) planar bilayer.^24^ After completion of this process, the value of Δ*f* was −24.3±0.5 Hz and Δ*D* was 0.04±0.1×10^−6^ indicating that a high-quality bilayer with a low number of defects was formed.24b VLP binding was monitored at 22 °C after inducing domain growth by heating the chamber to 50 °C (one hour) and cooling it down to 22 °C, in analogy to the AFM experiments. The binding reaction (started at *t*=0 in Figure 3) could be followed by a decrease in Δ*f* and an increase in Δ*D.* It reached saturation within 15 minutes and stabilized to a Δ*f* value of −8.7 Hz and Δ*D* at 0.85×10^−6^. To block potential defects in the bilayer, the sensor was incubated in bovine serum albumin (BSA) prior to addition of the VLPs.

**Figure 3:**
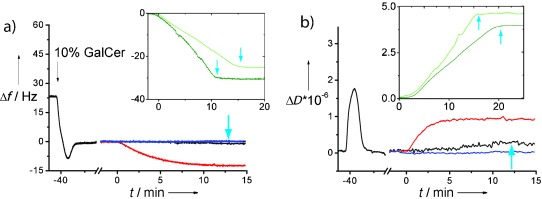
QCM-D study of norovirus binding to a bilayer containing 10 % GalCer. a) Frequency shifts Δ*f* and b) energy dissipation Δ*D* versus time *t* after addition of VLPs for unlabeled GalCer at 37 °C (black) and 22 °C (red), and for GalCer-Atto647N at 22 °C (blue). Bilayer formation (*t*<0) was followed by injection of the VLPs at *t*=0 (Δ*f* and Δ*D* were set to zero for *t*=0). Inserts: binding to a bilayer containing 5 % non-clustering H type 1 at 37 °C (light green) and at 22 °C (dark green). Blue arrows=rinsing steps.

The VLP binding was GSL-specific as confirmed by control experiments performed with pure POPC bilayers (−Δ*f*<0.7 Hz; data not shown). Furthermore, the results were confirmed with GalCer isolated from other sources and having monospecies ceramides as well as with bilayers containing different amounts of GalCer (see Supporting Information).

To investigate the influence of GSL clustering on VLP binding, experiments were also performed under experimental conditions where GalCer is in the fluid phase. At 37 °C, binding was reduced by one order of magnitude to values of Δ*f*=−1.2 Hz and Δ*D*=0.09×10^−6^, that is, close to the detection limit of the instrument, in agreement with the small (less than 0.4 %) domain fraction observed at 37 °C. Control experiments further confirm that the temperature does not negatively affect the activity of the VLPs (insert: Figure 3; see also Ref. 25 and Supporting Information, Figure S1). We further tested VLP binding to a bilayer containing 10 % GalCer labeled with the dye Atto647N by acyl-chain replacement. At room temperature, GalCer-Atto647N did not form domains in SLBs (see Supporting Information) and no binding of VLP was observed (Figure 3). This may be due to two reasons: GalCer-Atto647N does not by itself promote strong VLP binding when in the liquid phase, or it does not have the right conformation in the SLB owing to the introduction of the label (similar to GM1, in the context of cholera toxin^26^). Under the same experimental conditions, VLP binding to a bilayer containing 5 % H type 1, which is known not to form a gel-phase at room temperature (see Ref. 25) remained in the linear regime until rinsing at *t*=15 minutes, and saturation was not reached (Figure 3 a insert).6c In contrast, VLP binding to GalCer bilayers was saturated within ten minutes, suggesting that all accessible GalCer ligands become engaged in VLP binding. Because the total amount of GSL was larger for GalCer than H type 1, this indicates that only a fraction of the GalCer ligands is exposed in a way that favors VLP binding. Moreover, Δ*D* versus Δ*f* plots further indicate that the VLPs might locally reach a maximal coverage, as expected for cases when the VLPs are binding to well-defined areas on the bilayer. This is further discussed in the Supporting Information. Taken together, the QCM-D results suggest that the VLPs preferentially bind to GalCer in domains while, under these conditions, binding to H type 1 seems less dependent on the presence of preformed domains.

To gain further insight into domain recognition by the norovirus VLPs, we complemented our results with fluorescence microscopy experiments using bilayers further doped with 1 % (*w*/*w*) 2-[12-(7-nitrobenz-2-oxa-1,3-diazol-4-yl)amino]dodecanoyl-1-hexadecanoyl-*sn*-glycero-3-phosphocholine (NBD-PC). The fluorescent lipids were shown to predominantly partition into the gel-phase (Figure 4). At 10 % GalCer, the domains were too small to be resolved with optical microscopy. When the amount of GalCer was increased to 20 % or 35 %, domains were easily visualized by fluorescence microscopy as bright fractal features (Figure 4 b), while at temperatures above 37 °C, the bilayers were mostly homogeneous, in agreement with the AFM measurements (see Supporting Information for fluorescence microscopy images). Fluorescence recovery after photobleaching (FRAP) experiments confirm that the lipids in the domains are immobile (as expected for a gel-phase) while lipids around the domains are highly mobile (see Supporting Information). In all cases, the presence of GalCer leads to a reduced lipid diffusivity and increased immobile fraction (see Supporting Information). This is attributed to the presence of gel-phase domains which 1) act as diffusion obstacles to NBD-PC in the fluidic phase and 2) trap fluorescent lipids in the gel-phase (Figure 4).

**Figure 4:**
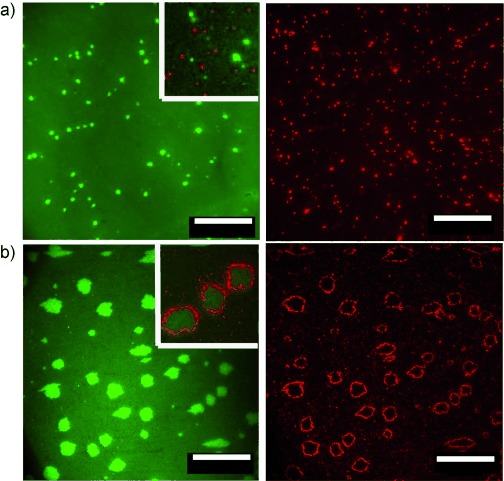
Fluorescence micrographs of NBD-containing (*λ*_ex_=460 nm/*λ*_em_=534 nm) GalCer domains (green/left) after addition of VLPs and rhodamine-labeled (*λ*_ex_=560 nm/*λ*_em_=580 nm) H type 1 vesicles (red/right) a) for 20 % GalCer and b) for 35 % GalCer bilayers. Scale bar=50 μm.

VLP binding was tested using bilayers containing 35 % (*w*/*w*) or 20 % GalCer and 1 % (*w*/*w*) NBD-PC. Such SLBs were then incubated with VLPs, and the site of binding was visualized by addition of rhodamine-labeled phospholipid vesicles containing H type 1 used as labels for the unstained VLPs (red in Figure 4). These vesicles are specific to norovirus VLPs, as shown previously,^27^ and were confirmed by control experiments performed in the absence of VLPs (data not shown).

Strikingly, for the 35 % GalCer bilayers, the VLPs recognized predominantly the edges and not the center of the GalCer domains. Some binding within the liquid phase was also observed and tentatively attributed to small GalCer clusters (Figure 4 b). This observation illustrates the need for an optimal degree of ligand spacing and presumably also lipid mobility to promote sufficiently strong multivalent attachment of the VLPs to GalCer bilayers. Ligand clustering may in fact favor the formation of multivalent interactions, but a too dense arrangement of the ligands can also lead to weakened binding owing to steric effects, as previously reported for cholera toxin binding to GM1.^28^ For 20 % GalCer domains, however, there was no correlation in binding between the VLPs and the edges of larger gel-phase domains (Figure 4 a). Rather, optimal GalCer presentation appears, in this case, to be associated with the presence of smaller (optically unresolved) features within the liquid phase. Even though these results may appear contradictory, they most likely illustrate an often ignored but crucial aspect of recognition events controlled by lipid bilayers. Multivalent interactions have delicate requirements with respect to how the ligands are presented. Subtle differences in GSL packing disorder, density, orientation, and spacing,^29^ which typically result from the formation of different lipid phases in bilayers are therefore likely to play a crucial role in determining the fate of the interaction and, in the case of virus binding, also infection.

Hence, while domain formation does not appear to be a requirement in the case of VLP binding to H type 1,^25^ we herein demonstrate that it is crucial for binding to GalCer. At 35 % GalCer, conditions for strong VLP binding appears at the rim of the domains, while at 20 % GalCer, binding occurs on domain features too small to be optically resolved. We concluded this using a combination of thin layer chromatography and in situ investigations on model membranes, which together provide unique opportunities to study GSL–pathogen interactions and allow us to consider essential parameters such as membrane organization, ligand presentation, and multivalency. These results could stimulate further research towards the identification of the physico-chemical properties of the membrane, which lead to the interesting contrasts reported herein. Especially, we believe that stimulated emission depletion microscopy^30^ will make it possible to further correlate lipid diffusion on the nanoscale with VLP binding.

The domain-dependent binding of norovirus GII.4 VLPs to GalCer, also sheds light on a potential new player in the norovirus infection process. We have earlier established that secretor (*FUT2*) gene-dependent glycosphingolipids bind norovirus, in excellent correlation to challenge and outbreak studies, showing the genetic linkage to disease resistance/susceptibility to most, but not all, of the human norovirus strains.^9^ GalCer is not only one of the major GSLs in human intestinal epithelial cells together with type 1 chain fucosylated GSL.10a, 19b, 31 It is also a co-receptor in HIV infection,11a where it has been proven responsible for transcytosis through intestinal epithelial cells.^32^ The observation that VLPs bind to both GSL types with different characteristics is thus of considerable interest when studying norovirus infection. When comparing GalCer to the other GSLs previously identified to bind to norovirus, it is worth noting that the carbohydrate head group of GalCer is shorter (it has only a single sugar unit) and therefore provides a possibility for intimate contact between the membrane and the virus capsid. Such GSLs with shorter headgroups may more often serve as receptors, owing to the close proximity with the bilayer membrane, facilitating internalization.11a The roles played by different GSLs may be altered owing to the local plasma membrane organization in different cell types and possibly at different stages of infection, from adhesion to egress. It is worth stressing, however, that the complexity of a real cell membrane and the possibility of non-equilibrium states induced by sophisticated cellular processes imply that model membranes may not permit a precise prediction of the cell membrane structures in vivo. However, as exemplified by the GalCer domains observed in our work, such studies set a basis for the physical origin of lipid domains and increase our understanding of their possible role in virus–host membrane interactions.

## Experimental Section

For the CBA,10a, 17a, 31 pure GSLs or GSL mixtures were applied to aluminum backed silica gel HPTLC plates developed in chloroform/methanol/water (60:35:8 by volume). After drying, plates were cut into sections, which were either stained chemically by anisaldehyde/sulfuric acid/acetic acid (1:2:97 by volume) or plasticized with poly(isobutyl methacrylate). The VLPs were added after blocking with BSA and detected after incubation with antiserum against norovirus and a secondary alkaline phosphatase-conjugated antibody.

The phospholipid vesicles for SLB formation were prepared by lipid-film hydration and extrusion through 30 nm polycarbonate membranes at 70 °C (22 °C if no GalCer was included). For fluorescence microscopy and AFM, the bilayers were formed on a clean glass substrate at 37 °C followed by extensive rinsing. AFM images were obtained in intermittent-contact mode in a temperature-stabilized liquid chamber. VLP binding was monitored in situ by QCM-D after formation of the bilayers on a SiO_2_ coated sensor and blocking with BSA. Fluorescence images were acquired at 60× magnification on an inverted fluorescence microscope equipped with a FITC and a TRITC filter cube. The vesicles used for VLP visualization were prepared as described above and contained 5 % H type 1, as well as 1 % 1,2-dihexadecanoyl-*sn*-glycero-3-phosphatidylethanolamine (rhodamine-DHPE).

## Notes

Detailed facts of importance to specialist readers are published as ”Supporting Information”. Such documents are peer-reviewed, but not copy-edited or typeset. They are made available as submitted by the authors.
