# Supplementary material for: Norovirus GII.4 Virus-like Particles Recognize Galactosylceramides in Domains of Planar Supported Lipid Bilayers
Source: Angew Chem Int Ed Engl. 2012 Oct 24;51(48):12020–4. doi: 10.1002/anie.201205972 (PMC3546384; doi:10.1002/anie.201205972)
Supplement: Supplementary file 1 [file anie0051-12020-SD1.pdf]

Angewandte Chemie
Eine Zeitschrift der Gesellschaft Deutscher Chemiker

Supporting Information
© Wiley-VCH 2012
69451 Weinheim, Germany

Norovirus GII.4 Virus-like Particles Recognize Galactosylceramides in Domains of Planar Supported Lipid Bilayers**

Marta Bally, Gustaf E. Rydell, Raphael Zahn, Waqas Nasir, Christian Eggeling, Michael E. Breimer, Lennart Svensson, Fredrik Höök, and Göran Larson**

anie_201205972_sm_miscellaneous_information.pdf

Experimental details

Materials

The norovirus VLPs used in this study were produced in SF-9 insect cells using the baculovirus construct from the Dijon 171/96 strain. The VLP has been described before and the baculovirus construct was a kind gift from Drs. P Pothier and E Kohli, Dijon, France.[1]

Glycosphingolipids (GalCer, histo-blood group Lewis a and H type 1 chain pentaglycosylceramides) were purified from human meconia, pooled according to ABO blood group, and characterized by mass spectrometry and ^{1}H-NMRspectroscopy.[2] Total non-acid glycosphingolipids from small and large intestines obtained after surgery, were prepared from epithelial cells obtained after a mild washing procedure [3] or as a mucosa scraping of the distal large intestine (case 5, in [4]). Individual glycolipid components were structurally characterized as above. The human tissue donors were typed for blood group ABO, Lewis and secretor status.[3b, 4] GalCer-Atto647N was produced as described in [5]. Native galactosylceramides, prepared from human brain, or GalCer with defined sphingosine(d18:1)-C16:0 or sphingosine–C24:1 ceramides were kind gifts from Prof. Jan Eric Månsson, Gothenburg, Sweden.[6]

Chromatogram Binding Assay

The original TLC chromatogram binding assay using radiolabelled Sendai virus was published 1984 [7] and was recently used for studies of norovirus.[2b] In short, GSLs (1-2 µg of pure GSLs and 10-20 µg for mixtures) were applied to aluminum backed silica gel 60 HPTLC plates (Merck, Darmstadt, Germany) and chromatographed with chloroform:methanol:water (60:35:8, by volume). Each GSL sample was applied at least twice on each TLC-plate. The plate was cut into sections, which were either chemically stained by anisaldehyde:sulfuric acid:acetic acid (1:2:97, by volume) or plasticized by dipping them in polyisobutylmethacrylate (P28, 0.3 % in hexan:diethylether (1:1, by volume)) and dried thoroughly. The plasticized plates were blocked by immersion in 3 % BSA, 0.05 % Tween-20 in PBS at room temperature (RT) for 1 h. Thereafter, VLPs (1 µg/mL in CBA dilution buffer (0.5 % BSA, 0.05 % Tween in PBS) were added to the surface and incubated for 2 h at RT. The plates were washed in CBA washing buffer (0.05 % Tween-20 in PBS), followed by addition of rabbit antiserum against norovirus (diluted 1: 3000 in CBA dilution buffer) to the plates, incubation for 1.5 h at RT and washed. Secondary antibodies (alkaline phosphatase conjugated goat anti-rabbit IgG, Sigma (St Louis, MO, USA)) were added (1:1000 in CBA dilution buffer). After washing, the staining was developed using Sigma Fast BCIP/NBT.

Lipid vesicle preparation

Vesicles containing 1-palmitoyl-2-oleoyl-*sn*-glycero-3-phosphocholine (POPC, Avanti Polar Lipids, USA) and GalCer or GalCer-Atto647N in appropriate amounts were prepared by lipid film hydration and extrusion. Briefly, the lipids were dried in a round-bottom flask under a gentle N_2 stream and in vacuum for at least 3 h. The so-obtained lipid film was then hydrated in pre-warmed TRIS buffer (10 mM TRIS, 100 mM NaCl, pH 7.0, 60 ^{o}C). Extrusion was performed at 70°C by pressing the mixture 11 times through a 30 nm polycarbonate membrane (Whatman, UK). The vesicle suspension was then cooled to room temperature and subsequently stored at 4 ^{o}C until further use. Fluorescent vesicles for microscopy studies were prepared with a similar procedure by adding 1 % (*w/w*) 2-(12-(7-nitrobenz-2-oxa-1,3-diazol-4-yl)amino)dodecanoyl-1-hexadecanoyl-*sn*-glycero-3-phosphocholine (NBD-PC, Avanti

lipids, USA) (ex/em:460/534) to the lipid mixture before solvent evaporation. The H type 1 vesicles used in QCM-D experiments contained 5 % (*w/w*) H type 1 glycosphingolipids and where extruded through a 30 nm polycarbonate membrane. The rhodamine vesicles used as labels for the microscopy experiment contained 5% H type 1 and 1% Lissamine- Rhodamine B-1,2-dihexadecanoyl-sn-glycero-3-phosphatidylethanolamine (rhodamine-DHPE, Invitrogen, USA) (ex/em:560/580) and were extruded through a 100 nm polycarbonate membrane. In both cases, hydration in TRIS buffer and extrusion were performed at room temperature.

Fluorescence microscopy

Fluorescence microscopy images were acquired using a Nikon Eclipse Ti-E inverted microscope and a 60X magnification (NA = 1.49) oil immersion objective (Nikon Corporation, Tokyo, Japan). The microscope was equipped with a mercury lamp (Intensilight C-HGFIE; Nikon Corporation), a FITC and TRITC filter cube (Nikon Corporation) and an Andor iXon+ EMCCD camera (Andor Technology, Belfast, Northern Ireland). Fluorescence recovery after photobleaching experiments were performed by bleaching circular areas with a diode pumped solid state laser at 475 nm (BWB-475-20E; B&W Tek Inc., Newark DE, USA).

Experiments were performed in glass-bottom microwells (96-well plate, MatTek Corporation, Ashland MA, USA) cleaned for at least 2 hours in 2% Hellmanex cleaning solution. The supported lipid bilayer was formed at 37 ^{o}C by addition of a pre warmed vesicle suspension (0.1 mg/ml, TRIS buffer, 30 min incubation) followed by extensive rinsing with warm buffer, taking care not to dry the surface at any time. Temperature treatments were performed in a laboratory stove and the samples were always imaged at 22 ^{o}C. The diffusion coefficient of fluorescent lipids was determined by fluorescence recovery after photobleaching experiments (FRAP). FRAP measures the lateral mobility of fluorescent lipids by bleaching the fluorescent tags in a well-defined circular area of the bilayer and monitoring lateral diffusion and healing of the bleached spot due to mixing of bleached and unbleached lipids.

The diffusion coefficient was determined using the Hankel transform method previously developed in our laboratory.[8] The diffusion coefficient and immobile fraction values are either the medians, when n=3, or the mean±standard deviation, when n=4. (n= number of individual experiments)

VLP binding experiments were performed by incubating the substrate 30 min with VLPs (66 μg/ml), followed by rinsing, addition of the rhodamine vesicles containing H type 1 (total lipid concentration: 30 μg/ml) and incubation for another 30 min. The sample was imaged after extensive rinsing.

AFM

Atomic force microscopy (AFM) images were acquired in intermittent contact mode with a Nanowizard I BioAFM (JPK Instruments, Berlin, Germany) using MikroMasch NSC 18/Cr-Au cantilevers (MikroMasch, Tallin, Estonia). Experiments were performed in a temperature-stabilized Biocell scanning chamber (JPK Instruments, Berlin, Germany) on glass coverslips. The image in Figure 2c was acquired on a Si Wafer (University wafers, south Boston, USA) mounted in a homemade scanning chamber. The substrates were cleaned for at least 2 hours in a 2 wt % sodium dodecyl sulfate (SDS) solution followed by a 30 min UV-ozone treatment.

Bilayers were formed at 37 °C with a procedure similar to the one described for fluorescence microscopy.

Height mode images were further processed using the SPM image processing software (JPK Instruments,Germany), the Scanning Probe Image Processor, SPIP (Image Metrology A/S, Denmark) and ImageJ (Image processing and analysis in Java, National Institute of Health; USA). The average domain coverage was estimated from images from two independent experiments. The total area imaged was 10^4 μm^2.

QCM-D experiments

Quartz crystal microbalance with dissipation monitoring (QCM-D) was carried out to monitor bilayer formation and VLP attachment in real time. This technique relies on monitoring the change of the fundamental resonance frequency and its overtones in thickness-shear modes upon mass adsorption on the surface of an oscillating quartz crystal.[9] A decrease in frequency corresponds to an increase in mass and thickness on the surface where the mass estimated from QCM-D includes the surrounding liquid associated with the oscillation. Additionally, QCM-D measures the damping of the oscillation which provides structural information as it can be related to changes in viscoelastic properties of the adlayer: the softer the film, the higher the ΔD values. For rigid films, this mass is directly proportional to the frequency shift (Δf).[10] For viscoelastic systems, the relationship is no longer linear and the adsorbed mass is obtained by applying the Voigt model to Δf and ΔD. [11] The QCM-D experiments were conducted with a Q-sense E4 instrument (Q-sense AB, Västra Frölunda, Sweden) equipped with 4 temperature-stabilized chambers. The SiO_2-coated QCM-D crystals were purchased from Q-Sense AB and cleaned overnight in 2 % (*w/w*) SDS and 45 min in a UV-ozone cleaner. All experiments were performed in TRIS buffer. After baseline stabilization the vesicle suspension was introduced in the measurement chamber at a flow rate of 15 μl/min. After completion of the bilayer formation process, the chamber was rinsed with buffer. GalCer bilayers were always formed at 37 °C, H type 1 bilayers at 22 °C. When applicable, the following temperature treatment was performed: 1 hour at 50 °C followed by cooling to 22 °C at a rate of 0.5 °C/min. The VLPs (8 μg/ml) were introduced in the measurement chamber under constant flow 50 (μl/min) and rinsed after 10 or 15 min. For GalCer bilayers, VLP injection was preceded by a 10 min blocking step with BSA (0.1 mg/ml). In the manuscript, we report the normalized frequency shifts ($\Delta f_5/5$) and energy dissipation (ΔD_5) of the 5th overtone. These values are either the medians, when n=3, or the mean±standard deviation, when n=6 (n= number of individual experiments).

Fluorescence microscopy

FRAP experiment were further performed in order to complemented our results with quantitative data on lipid mobility. In bilayers containing 10% GalCer, i.e. under conditions identical to those used for the AFM and QCM-D analyses, the diffusion coefficient of NBD-PC was 1.5 times lower than for NBD-PC in POPC (1.38±0.3 μm^{2}s^{-1} vs 1.99 μm^{2}s^{-1}) with an around two times higher immobile fraction (6.6±3.3% versus 3.5%). For comparison, gel-phase-free bilayers containing 5% H type1, had D= 1.82±0.3 μm^{2}s^{-1} and IF=1.16±1.46 μm^{2}s^{-1}. (See fig. S1.)

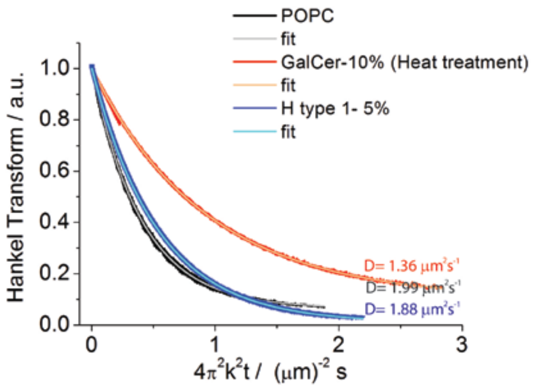

Fig. S1: FRAP experiment on bilayers containing 1% NBD-PC: Hankel transform of the fluorescence intensity of a bleached spot for a POPC bilayer (black), a bilayer containing 10% GalCer (red) and a bilayer containing 5% H type 1 (blue). All experiments were performed at room temperature (~21°C).

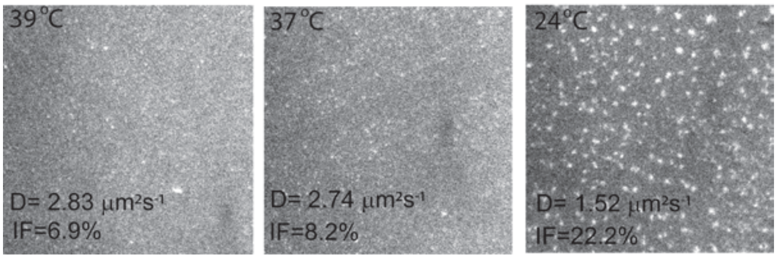

Fig. S2: Morphology, diffusion coefficient and immobile fraction of POPC bilayers containing 35% GalCer and 1% NBD-PC at 39°C, 37°C, and 24°C. Image: 100x100μm.

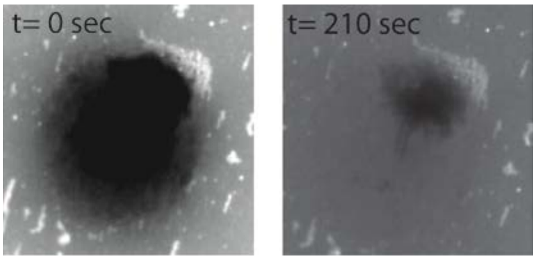

Fig. S3: FRAP on a large domain. The fluorescent lipids trapped in the GalCer gel phase are immobile while recovery is observed around the domains. Image: 65x65 μm. The experiment was performed at room temperature (~21°C)

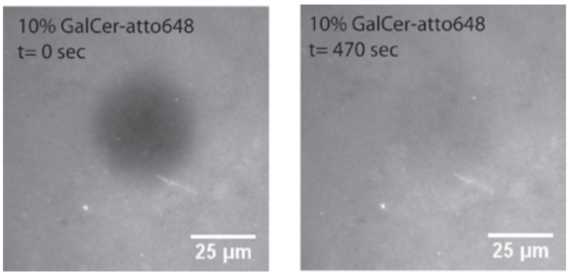

Fig. S4: FRAP on POPC bilayers containing 10% galactosylceramide 648N. The fluorescently labeled lipid is highly mobile within the bilayer. The experiment was performed at room temperature (~21°C).

Additional QCM-D data

Binding of norovirus VLPs to different GalCer types

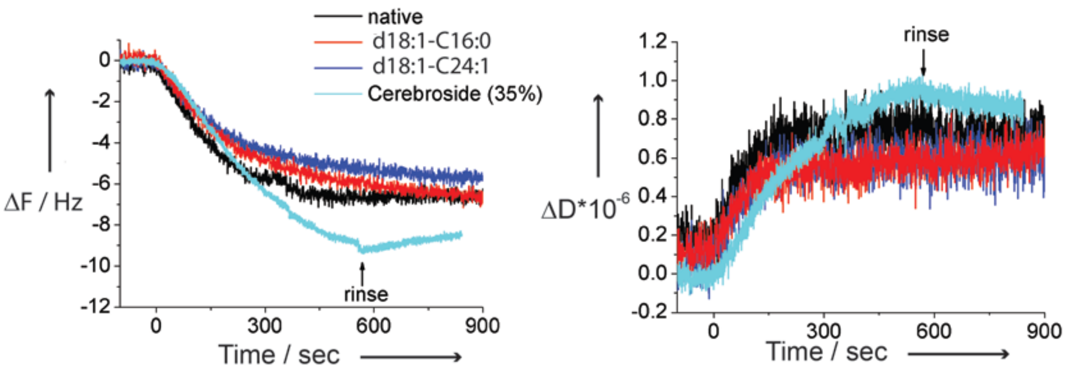

Fig. S5: QCM-D experiments showing the binding of VLPs to bilayers containing 10% native galactosylceramides (dark blue curve), 10% galactosylceramides with defined sphingosine (d18:1)-C16:0 ceramides (red curve), 10%GalCer with defined sphingosine–C24:1 ceramides (black curve) and 35% cerebrosides (Avanti lipids, USA) (light blue curve). Left: Frequency shift of the 5th overtone ($\Delta F_5/5$); right: corresponding energy dissipation (ΔD_5).

ΔD vs. ΔF plots

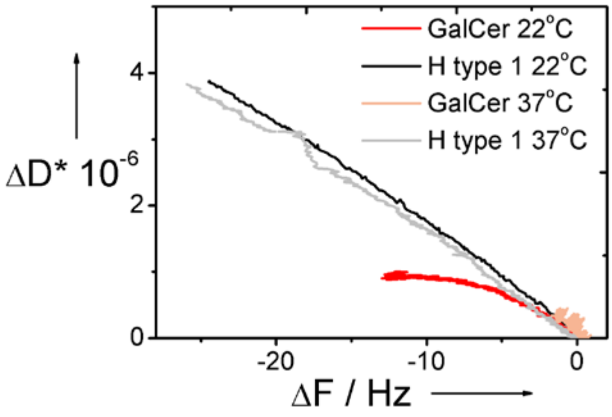

Fig. S6: A comparison of ΔD vs Δf for both GSL systems provides further insight on the binding behavior. For H type 1, VLP binding was essentially linear, while binding to GalCer deviated from the linear behavior for $\Delta f < -2$ Hz (Figure 3b). The successively lower ratio between ΔD to Δf is most likely attributed to a local crowding of VLPs since the amount of water mechanically coupled to the oscillation decreases when the surface is increasingly saturated with virus particles.[12] It cannot be excluded, however, that VLP deformation or penetration into the bilayer contributes to the observed deviation.

[1] B. Nicollier-Jamot, V. Pico, P. Pothier, E. Kohli, *J Clin Microbiol* **2003**, *41*, 3901-3904.

[2] aK. A. Karlsson, G. Larson, *J Biol Chem* **1981**, *256*, 3512-3524; bJ. Nilsson, G. E. Rydell, J. Le Pendu, G. Larson, *Glycoconjugate J* **2009**, *26*, 1171-1180.

[3] a) M. E. Breimer, G. C. Hansson, K. A. Karlsson, G. Larson, H. Leffler, *Glycobiology* **2012**, *in press*; b) S. Bjork, M. E. Breimer, G. C. Hansson, K. A. Karlsson, H. Leffler, *J Biol Chem* **1987**, *262*, 6758-6765; c) J. Holgersson, P. A. Jovall, M. E. Breimer, *J Biochem* **1991**, *110*, 120-131.

[4] J. Holgersson, N. Stromberg, M. E. Breimer, *Biochimie* **1988**, *70*, 1565-1574.

[5] V. Mueller, C. Ringemann, A. Honigmann, G. Schwarzmann, R. Medda, M. Leutenegger, S. Polyakova, V. N. Belov, S. W. Hell, C. Eggeling, *Biophys J* **2011**, *101*, 1651-1660.
[6] J. E. Mansson, L. Svennerholm, *Clin Chim Acta* **1982**, *126*, 127-133.
[7] G. C. Hansson, K. A. Karlsson, G. Larson, N. Stromberg, J. Thurin, C. Orvell, E. Norrby, *Febs Lett* **1984**, *170*, 15-18.
[8] P. Jonsson, M. P. Jonsson, J. O. Tegenfeldt, F. Hook, *Biophys J* **2008**, *95*, 5334-5348.
[9] a) M. Rodahl, F. Hook, A. Krozer, P. Brzezinski, B. Kasemo, *Rev Sci Instrum* **1995**, *66*, 3924-3930; b) M. Rodahl, B. Kasemo, *Rev Sci Instrum* **1996**, *67*, 3238-3241.
[10] G. Sauerbrey, *Z Phys* **1959**, *155*, 206-222.
[11] M. V. Voinova, M. Rodahl, M. Jonson, B. Kasemo, *Phys Scripta* **1999**, *59*, 391-396.
[12] P. Bingen, G. Wang, N. F. Steinmetz, M. Rodahl, R. P. Richter, *Anal Chem* **2008**, *80*, 8880-8890.
